# Congenital Heart Block and Its Association With Anti-Ro and Anti-La Antibodies in Pregnancy: A Case Report of a Rare Entity and a Review of the Current Evidence

**DOI:** 10.7759/cureus.45832

**Published:** 2023-09-23

**Authors:** Ioakeim Sapantzoglou, Zacharias Fasoulakis, George Daskalakis, Marianna Theodora, Panagiotis Antsaklis

**Affiliations:** 1 Obstetrics and Gynecology, Alexandra Hospital, University of Athens, Athens, GRC; 2 Obstetrics and Gynecology, University of Athens, Athens, GRC; 3 First Department of Obstetrics and Gynecology, National and Kapodistrian University of Athens, Athens, GRC; 4 First Department of Obstetrics and Gynecology, National and Kapodistrian University of Athens School of Medicine, Athens, GRC; 5 Obstetrics and Gynecology, National and Kapodistrian University of Athens, Athens, GRC

**Keywords:** adverse effects, fetal morbidity, antiphospholipid antibody syndrome (aps), congenital heart block, systemic lupus erythematosus

## Abstract

Systemic lupus erythematosus (SLE) is a heterogeneous chronic, multisystem, inflammatory autoimmune disorder with variable clinical features, with its manifestations being attributed to the presence of multiple autoantibodies and their subsequent autoimmune reactions. Multiple organs may be involved, with the kidneys, the joints, and the skin being the most common, increasing maternal and fetal morbidity and mortality. Our current article describes the case of a 32-year-old primigravida who was referred to our department after the detection of fetal bradycardia and the strong suspicion of an underlying cardiac abnormality. After a detailed fetal and maternal assessment, the diagnosis of SLE-associated fetal congenital heart block was established, and the appropriate management and treatment were provided, factors that led to the uncomplicated delivery and prompt successful management of an otherwise severely affected fetus. Our work, also, includes a detailed review of the accumulated evidence regarding the association between autoantibodies and congenital heart block, the available screening modalities of the condition, and its potential therapeutic interventions.

## Introduction

Systemic lupus erythematosus (SLE), a heterogeneous chronic, multisystem, inflammatory autoimmune disorder, is one of the most common connective tissue diseases with quite variable clinical features [1], with its manifestations being attributed to the presence of multiple autoantibodies and their subsequent autoimmune reactions. Multiple organs may be involved, with the kidneys, the joints, and the skin being the most common. The prevalence of the disease is reported to be 28 to 150 per 100,000 individuals, and given the fact that it is more common in women and young adults, pregnancy is not uncommon in affected women with approximately 3300 deliveries per year in the United States coming from mothers affected by SLE [2].

Maternal morbidity and mortality appear to increase in women with SLE who are at increased risk of thrombosis; thrombocytopenia; infection; multiorgan disease, namely nephritis and preeclampsia; and an overall 20-fold increase in maternal mortality [3]. In addition, some patients with SLE may also suffer from antiphospholipid syndrome (APS) which poses an increased risk for first-trimester losses and thrombosis. Furthermore, SLE can be associated with several fetal complications including preterm delivery, fetal growth restriction, stillbirth, and neonatal lupus, which is a rare but potentially fatal complication that might include skin lesions, congenital heart block (CHB), hepatitis, and hematologic abnormalities such as anemia and thrombocytopenia [4].

Our current article describes the case of a 32-year-old primigravida who was referred to our department for further management and follow-up after the detection of fetal bradycardia and the strong suspicion of an underlying cardiac abnormality. After a detailed fetal and maternal assessment, the diagnosis was established, and the appropriate management and treatment were provided, factors that led to the uncomplicated delivery and prompt successful management of an otherwise severely affected fetus. This article, also, entails a thorough review of the available data regarding the presence of autoantibodies and CHB, the currently available screening strategies, and the potential therapeutic approaches.

## Case presentation

A 32-year-old G1P0 (the woman who is pregnant for the first time and has not yet delivered) was referred to our department at 28 + 2 weeks of gestational age after her obstetrician detected severe fetal bradycardia (fetal heart rate [FHR] < 60 bpm) and an increase in the size of fetal right and left ventricles during a routine ultrasound assessment. Her medical history was unremarkable except for the diagnosis of juvenile idiopathic arthritis seven years ago, which has been in complete remission since then and after the initiation of hydroxychloroquine (200 mg twice daily). She was, also, receiving iron, calcium, vitamin D, and folic acid supplements as part of her pregnancy regimen.

The course of her pregnancy has been unremarkable so far. The first-trimester scan revealed a normal nuchal translucency measurement (NT:1.4 mm) as well as normal levels of b-human chorionic gonadotropin (bhCG) and pregnancy-associated plasma protein-A (PAPP-A) (bhCG:1.1MoMs, PAPP-A:1.3MoMs) with subsequent low risk for chromosomal abnormalities (T21:1/9050, T18:1/16782, T13:1/17772). The mid-gestation anatomy scan did not reveal any structural abnormalities; the estimated fetal weight (EFW) was on the 20th centile, and the FHR was 135 bpm. On the day prior to her referral, the ultrasound assessment calculated EFW to be on the ninth centile, and severe fetal bradycardia (FHR < 60 bpm) was noted. An emergency referral to a fetal cardiologist was made, and the fetal echocardiogram revealed a second-degree CHB (atrial rate: 130 bpm, ventricular rate: 55 bpm) and mild dilatation of the fetal cardiac ventricles, and a detailed rheumatologic assessment was suggested.

In our department, a full blood and immunological assessment was performed (Ht: 31.9%, Hb: 12.3 g/dl, WBC: 9800/μL, PLT: 588000/μL, D/Dimers: negative, C3/C4 complement assays: negative, serum glutamic-oxaloacetic transaminase [SGOT]: 14 U/L, serum glutamic pyruvic transaminase [SGPT]: 13 U/L, urea: 19 mg/dl, and Cr: 0.6 mg/dl). The immunological assays guided the investigation toward the diagnosis of SLE (antinuclear antibodies/ANA: positive [1/1280], anti-dsDNA: positive [17I U/ml], anti-Ro: positive [2,2U], anti-La: negative, anti-Sm: negative, and anti-cyclic citrullinated peptide [anti-CCP]: negative); the urinalysis revealed dysmorphic red blood cells and red blood cell casts, and the 24-hour urinary collection demonstrated the presence of 542 mg of protein. The ultrasound scan that was performed in our department confirmed a singleton pregnancy with no obvious extracardiac structural abnormalities with an EFW of 919 g (seventh centile) along with Doppler assay abnormalities (increased umbilical artery pulsatility index [PI] with positive end diastolic flow [EDF] and decreased PI of the middle cerebral artery [MCA]).

**Figure 1:**
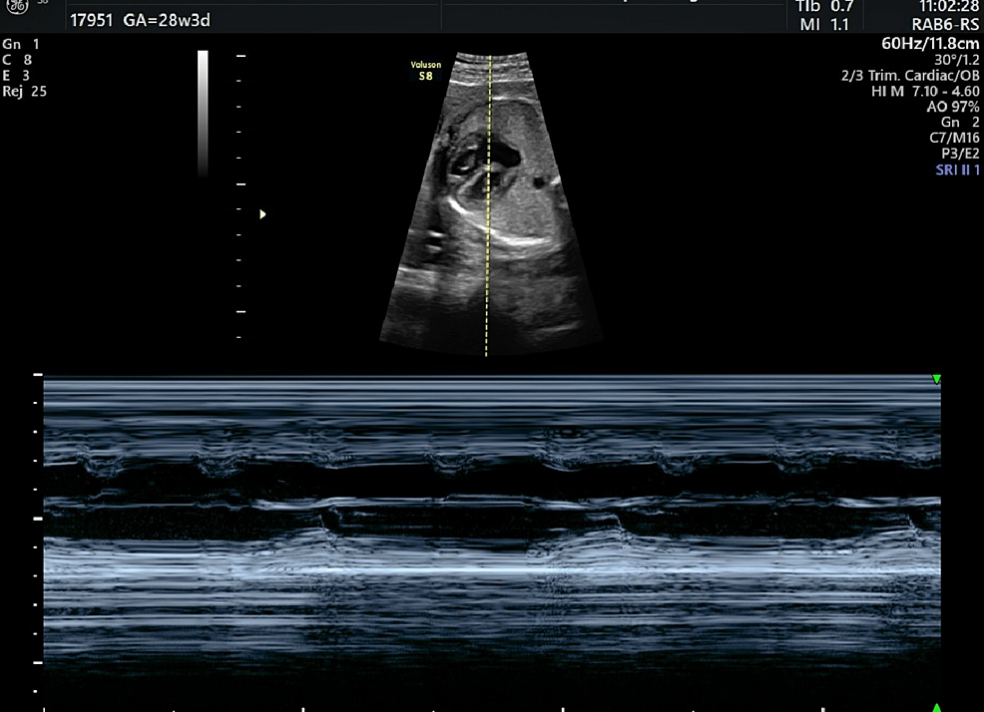
Detailed fetal echocardiography demonstrating the presence of second-degree heart block

**Figure 2:**
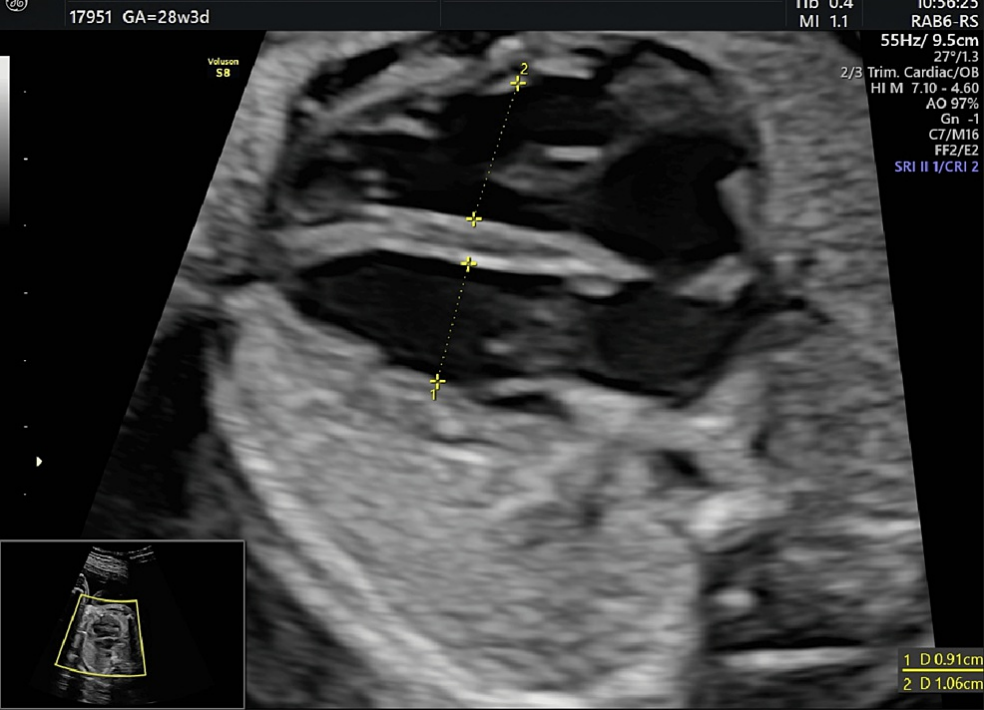
Detailed fetal echocardiography demonstrating the mildly dilated ventricles of the fetal heart

The fetal echocardiogram confirmed the presence of second-degree CHB (Figure 1 and Videos 1, 2) as well as the dilatation of the fetal cardiac ventricles (Figure 2). The combination of the above-mentioned findings led us to the newly established diagnosis of SLE, a growth-restricted fetus with a concomitant fetal cardiac conduction disorder due to the presence of anti-Ro antibodies. In conjunction with our rheumatology department, hydroxychloroquine was continued at the same dosage (200 mg twice daily), and dexamethasone was initiated (5 mg once daily). The pregnancy was closely monitored with cardiotocograms (CTGs) twice per day, ultrasound assessment of the fetal biometry once per week, and evaluation of the Doppler parameters and amniotic fluid every three days. The fetal bradycardia persisted in every ultrasound scan, and CTG was performed. Due to the progressive growth restriction and Doppler parameter deterioration, a cesarean section (CS) was performed at 33 + 1 weeks of gestational age (EFW: 1251 g [0.2th centile], absent EDF in the umbilical artery). A healthy female newborn was delivered with a gestational weight of 1500 g, APGAR scores of 7 and 10 at one and five minutes, respectively, a heart rate of 65 bpm, and normal umbilical cord gas parameters (pH: 7.27, pCO_2_: 52 mmHg, pO_2_: 27 mmHg, HCO_3_: 21 mmol/L, base excess in the extracellular fluid compartment [BEEcf]: 4.0 mmol/L). The newborn was immediately transferred to the local cardiothoracic hospital where an external pacemaker was initially applied, which was replaced by an internal pacemaker at 35 days of life. Currently, the toddler is 14 months old, healthy, of appropriate weight and growth, has reached all of her developmental milestones, and remains under close surveillance by pediatric cardiologists.

**Video 1:** Detailed fetal echocardiography demonstrating the presence of severe fetal bradycardia

**Video 2:** Detailed fetal echocardiography demonstrating the presence of severe fetal bradycardia: septal view of the fetal heart

## Discussion

SLE is a multisystem, inflammatory autoimmune disease characterized by the alternation of relapses and remissions. Several organs and tissues might get affected, a fact that poses an increased risk of maternal morbidity and mortality, and the initial diagnosis of the condition during pregnancy has been repeatedly associated with a rise in morbidity rates [3,4]. SLE has been associated by several well-designed studies with an increased risk of pregnancy loss (three-fold increase), preeclampsia (15%-35%), fetal growth restriction (6%-5%), and preterm delivery (19%-49%) [5]. Even though it was originally believed that pregnancy itself, due to the hormonal derangements it is accompanied with, may yield disease flares, several recent studies have demonstrated contradicting results. In terms of relapse severity, most flares over the course of pregnancy are quite mild, up to 30% might be severe, and only a few could pose a direct threat to the mother’s overall health. In terms of the timing, flares may take place during every trimester as well as in the postpartum period [4].

The most common organs affected include kidneys, skin, joints, heart, and brain with the most common symptoms being fatigue (80%-100%), fever (80%-100%), arthritis (80%-95%), myalgias (70%), weight loss (60%), and photosensitivity (60%) [3].

More specifically, lupus nephritis remains one of the most serious complications of SLE with one-third of the patients being affected at the time of diagnosis [6] and flares seem to be more common in women with active renal disease who become pregnant. The presence of renal disease makes the distinction between preeclampsia and lupus nephritis quite challenging, and even though several laboratory parameters have been proposed to facilitate such a distinction, none of those has been proven to be accurate [3]. Cutaneous lupus entails a group of skin disorders that may occur as a consequence of SLE or independently, with pregnancy outcomes being unaffected in the latter case. Hematologic disorders related to SLE include anemia, leukopenia, and thrombocytopenia, with anemia affecting roughly half of the patients and thrombocytopenia affecting approximately one-fourth of them [3]. Furthermore, while central nervous system (CNS) involvement might be rare, it includes significant manifestations such as seizures, neuropathy, psychosis, delirium, vasculitis, and diffuse cerebritis, which require prompt assessment, evaluation, and management [7], whereas joint manifestations, typically in the form of migratory, symmetrical arthritis, might affect the majority of the patients, with its frequency ranging from 69% to 95%.

**Figure 3:**
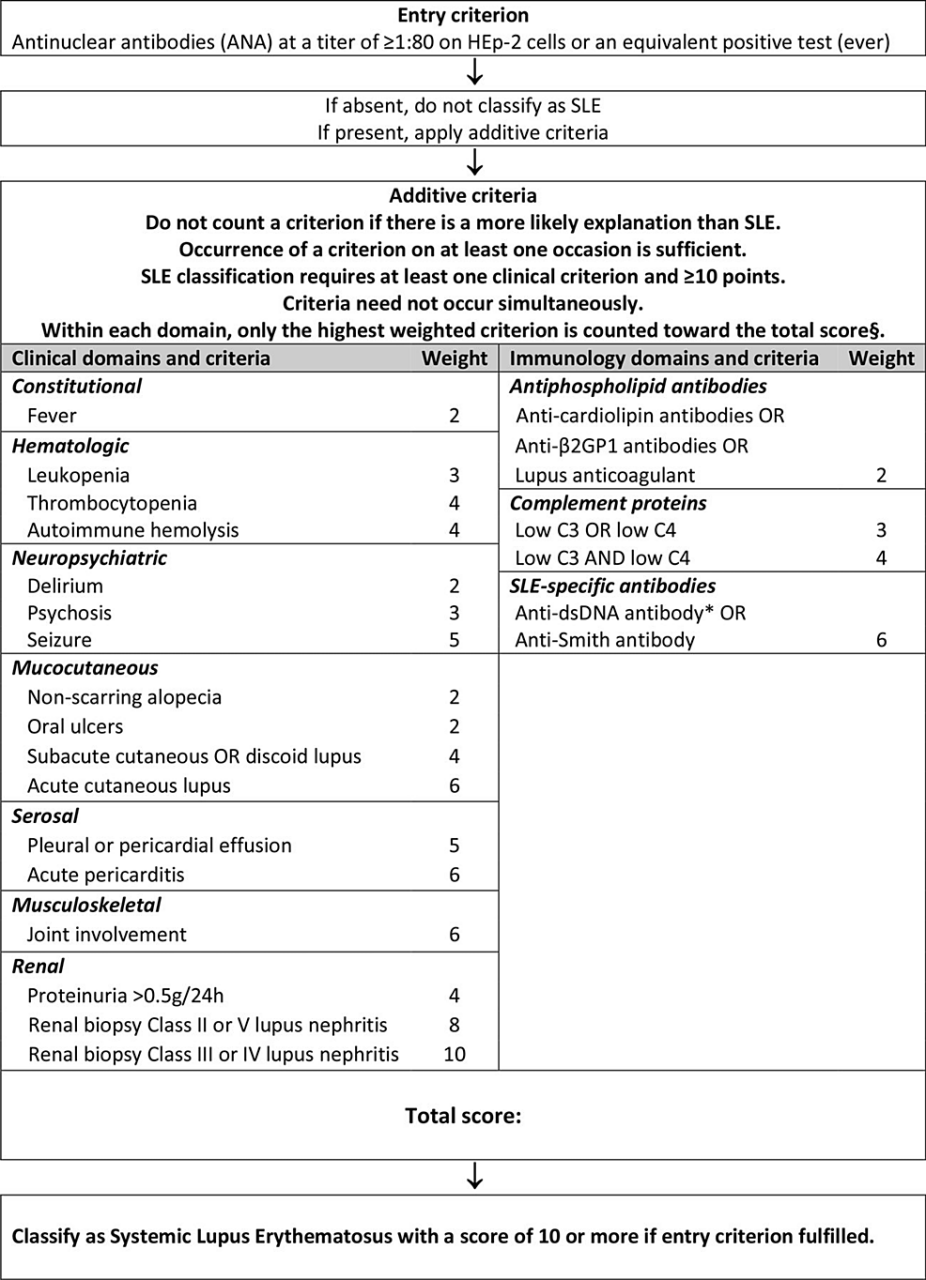
2019 EULAR/ACR classification criteria for systemic lupus erythematosus EULAR: European Alliance of Associations for Rheumatology; ACR: American College of Rheumatology; anti-b2GP1: Anti-beta-2-glycoprotein-1; anti-dsDNA: Antidouble-stranded DNA; SLE: Systemic lupus erythematosus. Source: Ref. [8].

To our days, the diagnosis of SLE remains a challenging task due to its wide range of clinical features and the absence of pathognomonic laboratory findings. Recently, the European Alliance of Associations for Rheumatology (EULAR)/American College of Rheumatology (ACR) published a classification system using antinuclear antibodies (ANA) as an entry criterion combining them with hierarchically clustered clinical, histological, and laboratory criteria in order to develop a data-driven diagnostic approach of SLE (Figure 3) [8]. Such an approach was initially developed for research purposes, but it was quickly implemented in the clinical practice carrying a sensitivity of 96% and a specificity of 93%.

At this point, it is necessary to highlight that fetuses are not only affected by the prematurity associated with SLE, either spontaneous or iatrogenic (due to preeclampsia or deterioration of maternal health), but also by neonatal lupus erythematosus (NLE), as demonstrated in our case report. NLE complicates one in 20,000 live births, and it consists of a serious condition that entails a highly variable clinical presentation, including skin lesions, CHB, hepatitis, anemia, and thrombocytopenia. It should be noted that most, but not all, of the NLE-affected neonates are born to women with SLE. Its pathophysiology is attributed to the transplacental passage of maternal IgG autoantibodies (anti-SSA and less commonly anti-SSB) that bind to cytoplasmic ribonucleoproteins. Anti-SSA antibodies are present in roughly 30% of women with SLE, while anti-SSB antibodies are isolated in 15%-20% of them [9], and they have been associated with a 2% risk of CHB and a recurrence risk of 15%-20% in case of a previously affected fetus or newborn [7]. CHB includes a spectrum of conditions characterized by a progressive interruption of the electronic impulse conduction from the atria to the ventricles. It consists of three degrees of atrioventricular (AV) block, with the first degree characterized by a prolongation of the AV interval, the second degree by the conduction of some beats and the blockage of others, and the third degree by the interruption of the AV conduction which is complete in a way that the atria and the ventricles function independently. In view of the morbidity associated with the cardiac complications of those antibodies, several researchers and clinicians have assessed different screening strategies, preventive modalities, and therapeutic interventions, but the evidence remains contradicting.

To begin with, in view of the screening strategy, Ramos-Casals et al. in 2019 suggested ultrasound and home Doppler monitoring of pregnancies with anti-SSA autoantibodies through the EULAR recommendations [10]. In accordance with the above, the ACR in their Reproductive Health Guideline in 2020 [11] also recommended serial fetal echocardiography starting from 16 to 18 weeks of gestational age and continuing up to week 26 due to the fact that this is the most common gestational period for the fetuses to develop CHB. However, in the recently published Society for Maternal-Fetal Medicine (SMFM) Consensus Statement #64 [12], the authors remain skeptical of the benefit of such a screening modality, consensus that was based on the still unproven effectiveness of the available therapeutic interventions [13]. Their rationale was the result of the published data of a number of studies that should be, however, analyzed with caution. PR interval and Dexamethasone Evaluation (PRIDE) study enrolled 127 pregnant women with anti-SSA antibodies (with or without anti-SSB antibodies) and followed them up with weekly ultrasound scans from 16 to 26 weeks of gestational age and biweekly thereafter, with dexamethasone administered to those with first- or second-degree heart block. The treatment efficacy could not be proven as some cases were reversed while others progressed, and several cases of third-degree block occurred without a progression through the two milder heart block degrees. However, the authors concluded that intense monitoring of such cases seems appropriate for the early identification of potential reversible injury, such as PR interval >150 msec.

In view of the treatment options, biologic evidence suggests that anti-inflammatory agents such as steroids and immunoglobulins (IVIG) as well as beta-mimetics may potentially reduce the inflammation and the subsequent scarring attributed to the causative autoantibodies. Current evidence remains conflicting, and the extraction of any results is still under question. Eliasson et al. [14], in their retrospective study, did not manage to report any significant effect of corticosteroids on congenital second- and third-degree heart blocks, and their results were aligned with more recent data that demonstrated no significant prevention of the disease progression, pacemaker implantation, and reduction of mortality in a cohort of fetuses that were administered with steroids within one week of CHB detection [15]. Furthermore, a meta-analysis performed by Ciardulli et al., which included a total of 71 fetuses with second-degree heart block who received treatment with steroids and assessed the rate of progression to complete heart block, did not manage to demonstrate any statistically significant benefit, being limited by the small sample size of the included studies. The addition of other medications to corticosteroids such as IVIG or beta-mimetics showed no differences, or the better neonatal outcomes could not be appreciated due to the lack of a control group or the inability to adjust for potential confounding factors [16,17]. Contradictory to the above and at odds with the SMFM statement, a number of recent studies have demonstrated encouraging results, and a number of experts have been critical of the statement [18]. The rationale of the criticism lies in the fact that the success of the treatment should not only be limited to the nonprogression of first- and second-degree heart blocks to complete heart block but should be extended to the overall fetal and postnatal survival. As such, Cuneo et al. demonstrated that treatment of first- and second-degree AVB with steroids has prevented its progression to third-degree AVD and even restored the sinus rhythm in some cases [19], while Mawad et al. revealed a decrease in the rate of dilated myocardiopathy and an increase of fetal, neonatal, and one-year survival of fetuses with complete heart block who received prompt treatment [17]. In the meta-analysis performed by Ciardulli et al., the authors opined collectively that the use of steroids should not be discouraged, irrespective of the inconclusive results of their study [16]. Finally, Trucco et al. showed, in their multicenter retrospective cohort, an 80% patient survival, all with normal ventricular function, a finding that is even more encouraging when it is compared with historic cohorts in which the mortality rate was reported to be as high as 78% [20].

Based on the published data, it is obvious that the optimal management and treatment of fetuses affected by the transplacental passage of anti-SSA and anti-SSB autoantibodies remain under question. At the moment, two major studies are ongoing that will hopefully guide our approach in an evidence-based manner. The first (STOP BLOQ: Surveillance and Treatment to Prevent Fetal Atrioventricular Block Likely to Occur Quickly; ClinicalTrials.gov identifier: NCT04474223) is a prospective study that stratifies patients according to their antibody titers, implements tight home Doppler surveillance in the high-titer cohort, and investigates the effectiveness of IVIG and steroid treatment on the restoration of sinus rhythm and the prevention of progression on fetuses affected by second-degree heart block. The second (Slow Heart Registry of Fetal Immune-Mediated High-Degree Heart Block; ClinicalTrials.gov identifier: NCT04559425) is prospectively designed to assess the morbidity and mortality of treated and non-treated fetuses over the first two years of their lives. Given the conflicting evidence so far, their results seem necessary for solid guidelines to be developed.

## Conclusions

Fetal AV block develops in approximately 2% of mothers with anti-SSA autoantibodies and, although uncommon, it carries significant mortality and morbidity. Current evidence regarding the screening and the ultimate management of the condition are rather conflicting and, at the moment, are based on small-sized and underpowered observational studies. Nevertheless, it is reasonable to conclude that serial fetal echocardiography starting from 16 weeks of gestational age and continuing up to week 26 should be offered in those high-risk patients. However, it should be noted that the currently limited data yield the need for further research and well-designed studies in an effort for the optimal approach to be modeled.
